# Transforming growth factor-β_1_ induces connective tissue growth factor expression and promotes peritoneal metastasis of gastric cancer

**DOI:** 10.1042/BSR20201501

**Published:** 2020-09-18

**Authors:** Ling Lv, Fu-Rong Liu, Di Na, Hui-Mian Xu, Zhen-Ning Wang, Cheng-Gang Jiang

**Affiliations:** 1 Department of Thoracic Surgery, The First Affiliated Hospital of China Medical University, Shenyang, Liaoning Province, China; 2 Department of Cell Biology, China Medical University, Shenyang, Liaoning Province, China; 3 Department of Surgical Oncology and General Surgery, Key Laboratory of Precision Diagnosis and Treatment of Gastrointestinal Tumors, Ministry of Education, The First Affiliated Hospital of China Medical University, Shenyang, Liaoning Province, China

**Keywords:** CTGF, Peritoneal metastasis, Stomach neoplasms, TGF-β1

## Abstract

Transforming growth factor-β_1_ (TGF-β_1_) is involved in human cancer development and progression. Nonetheless, the role of TGF-β_1_ as regards peritoneal metastasis of gastric cancer has not been completely characterized. In the present study, we investigated the exact role of TGF-β_1_ on peritoneal metastasis of gastric cancer. The results indicated that human peritoneal mesothelial cells (HPMCs) exposed to TGF-β_1_ or serum-free conditional medium (SF-CM) of SGC7901 that produced a large amount of TGF-β_1_ became exfoliated, apoptosis and exhibited signs of injury, and the tumor-mesothelial cell adhesion significantly increased. Connective tissue growth factor (CTGF) expression was also increased when HPMCs were exposed to TGF-β_1_ or SF-CM of SGC7901. However, these effects were significantly decreased when HPMCs were exposed to SF-CM of SGC7901-TGFβS, a TGF-β_1_ knockdown stable cell line. Animal studies revealed that nude mice injected with SGC7901-TGFβS cells featured a smaller number of peritoneal seeding nodules and lower expression of CTGF in ascites than the control cell lines. These findings suggest that TGF-β_1_ promotes peritoneal metastasis of gastric cancer and induces CTGF expression. Therefore, blockage of TGF-β_1_ or TGF-β_1_ signaling pathway might prevent and treat peritoneal metastasis of gastric cancer.

## Introduction

Despite significant advances in cancer research, cancer remains a worldwide health problem and mortality due to cancer remains high. Gastric cancer remains the fifth most prevalent cancer worldwide and the third leading cause of global cancer mortality [1]. There appears to be a decreasing trend in occurrence, notably in Western countries; it is still commonly reported in China and Japan. Even though the prognosis of patients with advanced gastric cancer seems to have improved as a result of the standardization of surgical techniques and recent advances in chemotherapy, the 5-year postoperative survival rate remains low [2]. Peritoneal metastasis is the most common and significant cause of mortality after surgery for gastric cancer [3]. However, the mechanisms of peritoneal metastasis have not been clearly defined.

Transforming growth factor-β (TGF-β) is a multifunctional cytokine, which influences cell differentiation, proliferation, motility and apoptosis [4]. Among the TGF-β family, which comprises TGF-β_1_, -β_2_ and-β_3_; TGF-β_1_ is most abundantly expressed, especially in various pathological conditions including chronic inflammatory disease and cancer [5–7]. To date, TGF-β_1_ has been identified as a double-edged sword in the process of human cancer, as it inhibits the development of tumorigenesis in the early stages and stimulates tumor growth as the tumor progresses [8,9]. In clinical studies, it was reported that an elevated serum level and overexpression of TGF-β_1_ in primary gastric cancer were significantly correlated with lymph node metastasis and poor prognosis in patients with gastric cancer [10]. However, whether TGF-β_1_ influences the peritoneal metastasis of gastric cancer remains unknown.

Connective tissue growth factor (CTGF), also known as CCN_2_, is a member of the CCN family, including cysteine-rich protein 61 (Cyr61), also known as CCN1, and nephroblastoma-over expressed gene (Nov), also known as CCN3, as well as Wisp-1/elm1 (CCN4), Wisp-2/rcop1 (CCN5) and Wisp-3 (CCN6) [11]. CTGF is believed to be a multifunctional signaling modulator involved in a wide variety of biologic or pathologic processes, such as angiogenesis, osteogenesis, fibrosis in kidneys and skin and tumor development [12,13]. It was reported that CTGF plays an important role in the progression of several types of cancer [14,15]. Although some studies have shown that CTGF acts as a downstream mediator of some of the effects of TGF-β_1_ on cell proliferation, migration, adhesion and matrix production [16,17], the potential interaction between these two factors in the progression of peritoneal metastasis has not been examined. Therefore, the purpose of this study was to: (1) determine the exact role of TGF-β_1_ on peritoneal metastasis of gastric cancer and (2) investigate the relationship between TGF-β_1_ stimulation and the expression of CTGF in peritoneal mesothelial cells.

## Materials and methods

### Reagents

Propidium iodide (PI) and trypsin were purchased from Sigma (St. Louis, MO, U.S.A.). DMEM, streptomycin and other cell culture supplies were from GIBCOBRL (Grand Island, NY, U.S.A.). Fetal bovine serum was from Hyclone (Logan, UT, U.S.A.). Acridine orange (AO) was obtained from Fluka (Ronkonkoma, NY, U.S.A.). 5(6)-carboxy fluorescein diacetate succinimidyl ester (CFSE) was from Molecular Probes (Eugene, OR, U.S.A.). Recombinant human TGF-β_1_, TGF-β_1_ and CTGF ELISA kits were purchased from R&D (Minneapolis, MN, U.S.A.). Trizol and Lipofectamine 2000 were purchased from Invitrogen (Carlsbad, CA, U.S.A.). SYBR^@^Primescript^TM^ RT-PCR kit was from Takara Biotechnology, Japan. CTGF and TGF-β_1_ primary antibody, as well as second antibody goat anti-mouse IgG and Rhodamine (TRITC)-conjugated affinipure goat anti-mouse IgG were obtained from Santa Cruz Biotechnology (Santa Cruz, CA, U.S.A.).

### Cell culture and treatment

Human peritoneal mesothelial cell line HMrSV5 was kindly provided by Prof. Pierre RONCO, Hospital TENON (Paris, France). HMrSV5 was originally isolated from human omentum. Human gastric cancer cell lines, MKN-45, MKN-1, AGS, SGC7901, BGC823 and MGC803 were obtained from the Department of Cell Biology, China Medical University, China. They were cultured in DMEM containing 10% fetal bovine serum, 100 U/ml of penicillin, 100 µg/ml of streptomycin at 37°C in a humidified atmosphere of 5% CO_2_. The cells were dislodged using 0.25% trypsin and 0.02 mol/l EDTA in PBS for subculture.

Before each treatment, human peritoneal mesothelial cells (HPMCs) were cultured in serum-free medium overnight. The cells were then treated with various concentrations of TGF-β_1_ (1, 5, 50 ng/ml) or serum-free conditional medium (SF-CM) from gastric cancer cells. HPMCs were incubated in the presence of TGF-β_1_ or SF-CM from gastric cancer cells for 6, 12, 24 and 48 h. After incubation, cells were examined under a phase-contrast microscope for alterations in size, shape and integrity of cell membrane, cytoplasm and nucleus. The culture media were harvested, and the cell layer was either harvested for flow cytometry study or washed and lysed for RNA extraction.

### Preparation of serum free conditional medium (SF-CM)

A total of 3 × 10^5^ cells were seeded in 100-mm tissue culture dish with regular medium for 2 days. Then, the cells were washed twice with PBS and incubated with 3 ml of serum-free DMEM. Two days later, the SF-CM was collected and centrifuged at 2000 ***g*** for 5 min, passed through filters (pore size, 0.45 µm) and stored at −80°C until use.

### Construction of TGF-β_1_ knockdown stable cell line

The small interfering RNA (siRNA) oligonucleotide was synthesized to target 5′-GCAGAGTACACACAGCATA-3′ in human TGF-β_1_ CDNA. Scramble siRNA was used as negative control. They were cloned into the siRNA expression vector pcPURβicassette (Takara), containing selective marker puromycin to facilitate selection of stable transfected cells. Stable cell lines were made by transfection of sipcPURβicassette- TGF-β_1_ or sipcPURβicassette-scramble into SGC7901 cells using Lipofectamine 2000. The cells were screened with puromycin (1.25 μg/ml), and the colonies were picked after 3 weeks, determined by RT-QPCR and Western blot. The expanded cells were then used for subsequent studies. Cells transfected with TGF-β_1_ siRNA or scramble siRNA were designated SGC7901-TGFβS cells or SGC7901-NC cells.

### Western blot analysis

Cells were lysed in RIPA buffer supplemented with protease inhibitor mixture for 30 min at 4°C. The cell lysates were then sonicated briefly and centrifuged (14,000 ***g*** at 4°C) for 15 min to remove insoluble materials. Equal amounts of protein were separated by SDS-PAGE and transferred to a PVDF membrane. Membranes were blocked with 5% nonfat dry milk and then incubated with first antibody, followed by horseradish peroxidase-conjugated secondary antibody. Protein bands were visualized by ECL chemiluminescence method.

### Enzyme-linked immunoassay (ELISA)

The levels of TGF-β_1_ in the SF-CM from gastric cancer cell lines and CTGF in the cultured media from treated HPMCs were measured using human Quantikine ELISA kits following the manufacturer’s instructions.

### Immunofluorescence and confocal imaging

The treated HPMCs on Lab-Tek tissue culture chamber slides were fixed in cold 100% methanol for 10 min, and then blocked with normal goat serum for 30 min. The cells were incubated with the primary antibody overnight at 4°C, washed three times in PBT (PBS with 1% Triton X-100), and then incubated with second antibody conjugated with Rhodamine. The DNA dye DAPI was used to stain the DNA. Cells were imaged on a Leica SP2AOBS confocal microscope.

### Real-time quantitative polymerase chain reaction (RT-QPCR)

Total RNA was isolated from cell pellets using Trizol reagent. Total RNA (1 µg) was converted to CDNA using a RT (reverse transcriptase) reaction kit. Real-time PCR was performed using Mx3000P real-time PCR system according to the manufacturer’s instruction and SYBR® Premix ExTaq as a DNA specific fluorescent dye. PCR was carried out for 40 cycles of 95°C for 5 s and 60°C for 40 s. The threshold cycle (*C*_t_) was obtained and relative quantities were determined for each sample normalized to GAPDH. Expressions of mRNA were calculated using the ΔΔ*C*_t_ method [18].

### Detection of apoptosis

For the treated human peritoneal mesothelial cells, apoptosis was quantified by the following two methods. (1) Flow cytometric analysis. Briefly, the attached and floating cells were mixed and washed with PBS. The cells were centrifuged, and the pellets were resuspended with 5 ml of cold 70% ethanol and fixed overnight at 4°C. The fixed cells were washed twice in PBS. Then, 50 µl of RNase (10 µg/ml) and 25 µl of propidium iodide (1 mg/ml) were added to the cells for 30 min at room temperature in dark. Flow cytometric analysis was performed with a FACS Caliber (FACScalibur; Becton Dickinson, U.S.A.). For each analysis, 10,000 events were collected and analyzed with CellQuest software. (2) Acridine orange staining. Briefly, cells were washed with ice-cold PBS, fixed with 4% paraformaldehyde for 10 min at room temperature, washed again with PBS, stained with 200 µl of acridine orange solution (10 µg/ml) and incubated in the dark for 5 min. The slides were rinsed briefly with PBS to remove unbound dye, mounted with nail polish and viewed under fluorescence microscope.

### Cell adhesion assay

HPMCs were plated at density of 1 × 10^4^ cells/well in collagen-coated 96-well plates. After incubation overnight at 37°C, HPMCs were incubated with different concentrations of TGF-β_1_, SF-CM from gastric cancer cells SGC7901, SGC7901-NC or SGC7901-TGFβS for 24 h. Then the gastric cancer cells BGC823, SGC7901 and MGC803 were prelabeled with CFSE by the method of van Kessel [19]. The CFSE-labeled cells (1 × 10^5^ cells/100 µl/well) were suspended in medium supplemented with 10% FBS and placed in 96-well plates containing HPMCs, and incubated at 37°C for 2 h. Dishes were then washed three times to remove non-adherent cells; the adherence of CFSE-labeled gastric cancer cells was determined by measuring the fluorescence using the fluorescent plate reader (Tecan GENios, Austria) at an excitation at 485 nm and emission at 530 nm. Cell adhesion was calculated as follows: % cell adhesion = mean fluorescence intensity of experimental wells/ mean fluorescence intensity of total cells plated ×100%.

### Animal experiment

This experiment was conducted in accordance with the guideline issued by the State Food and Drug Administration (SFDA of China). The animals were housed and cared for in accordance with the guidelines established by the National Science Council of Republic China.

All the animals’ experiments were done at the Department of Laboratory Animals of China Medical University and approved by the Institutional Animal Ethics Committee of China Medical University. Female BALB/c nude mice, 35–40 days old and weighing 20–22 g, were supplied by Shanghai Slac Laboratory Animal Limited Company. The mice were kept under sterile conditions and fed a sterilized mouse diet and water. Animals were anaesthetized via inhalation of isoflurance and a tumor cell suspension of 1 × 10^7^ cells in 0.5 ml DMEM were inoculated into the abdominal cavity of test mice. Three groups of mice were tested. Group 1 was inoculated with SGC7901 cells alone; group 2 was inoculated with SGC7901 cells stably transfected with sipcPURβicassette-scramble (SGC7901-NC) and group 3 was inoculated with SGC7901 cells stably transfected with sipcPURβicassette-TGF-β_1_ (SGC7901-TGFβS). The mice were killed by anesthesia overdose 7 weeks later, and any disseminated nodules present on the mesentery and diaphragm were evaluated. Ascites was collected directly from the peritoneal cavity. The cell-free ascites was obtained by centrifugation at 5000 ***g*** for 10 min at 4°C. Analysis of CTGF in ascites was performed using ELISA method according to the manufacturer’s instructions.

### Statistical analysis

All values in the text and figures are presented as mean ± SD. In univariate analysis, two-tailed χ^2^ tests for categorical variables and two-tailed *t* test for continuous variables were used for statistical comparisons. Values of *P*<0.05 were taken to show a significant difference between means.

## Results

### TGF-β_1_ concentration in serum-free conditional medium of gastric cancer cells and siRNA-mediated silence

**Figure 1:**
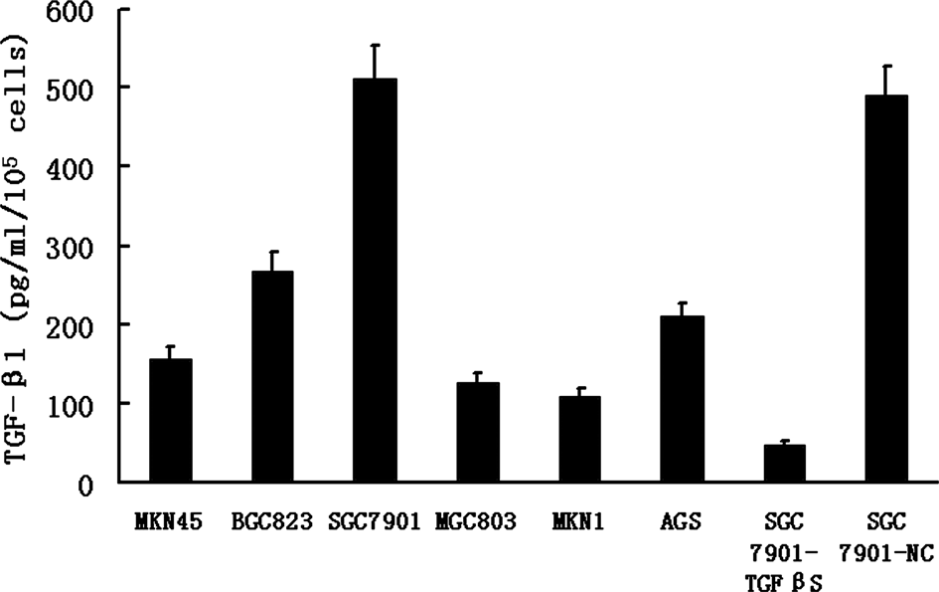
TGF-β_1_ concentration in SF-CM of various gastric cancer cells TGF-β_1_ in SF-CM of six gastric cancer cells and stable transfected cells was analyzed by ELISA. Cells’ number and supernatant volumes were measured when SF-CM were collected. Following this, the levels of TGF-β_1_ were statistically analyzed. Each column represents the mean ± SD of data from three experiments.

First, we examined the level of TGF-β_1_ in culture supernatants of various gastric cancer cells. As shown in Figure 1, the levels of secreted TGF-β_1_ in gastric cancer cell lines varied between 109 pg/ml/10^5^ cells and 512 pg/ml/10^5^ cells. SGC7901 produced the largest amount of TGF-β_1_ in the six gastric cancer cell lines. Therefore, we selected SGC7901 to construct TGF-β_1_ knockdown stable cell line and collect the SF-CM as stimulators of HPMCs.

**Figure 2:**
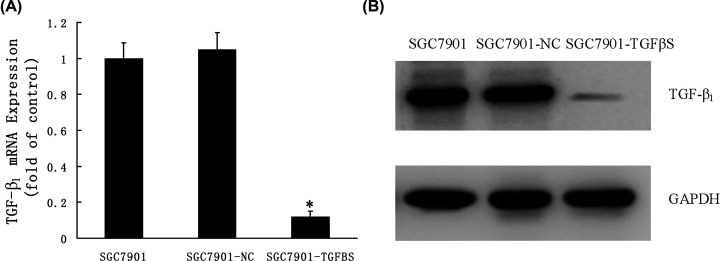
Expression of TGF-β_1_ in TGF-β_1_ knockdown stable cell line (**A**) RT-QPCR showing TGF-β_1_ mRNA levels in SGC7901, SGC7901-NC and TGF-β_1_ knockdown stable cell line SGC7901-TGFβS. Data are expressed as a fold change relative to control (control is SGC7901). Values are given as mean ± SD of three experiments. *P<0.05 as compared with control. (**B**) Western blot analysis of TGF-β_1_ protein expression in SGC7901, SGC7901-NC and TGF-β_1_ knockdown stable cell line SGC7901-TGFβS.

As shown, the level of TGF-β_1_ in culture supernatants was significantly decreased in the TGF-β_1_ knockdown stable cell line SGC7901-TGFβS as compared with SGC7901 or SGC7901-NC. RT-QPCR and Western blot also showed that expression of TGF-β_1_ markedly decreased in the TGF-β_1_ knockdown stable cell line SGC7901-TGFβS (Figure 2A,B).

### TGF-β_1_ increased CTGF concentration in serum-free conditional medium

**Figure 3:**
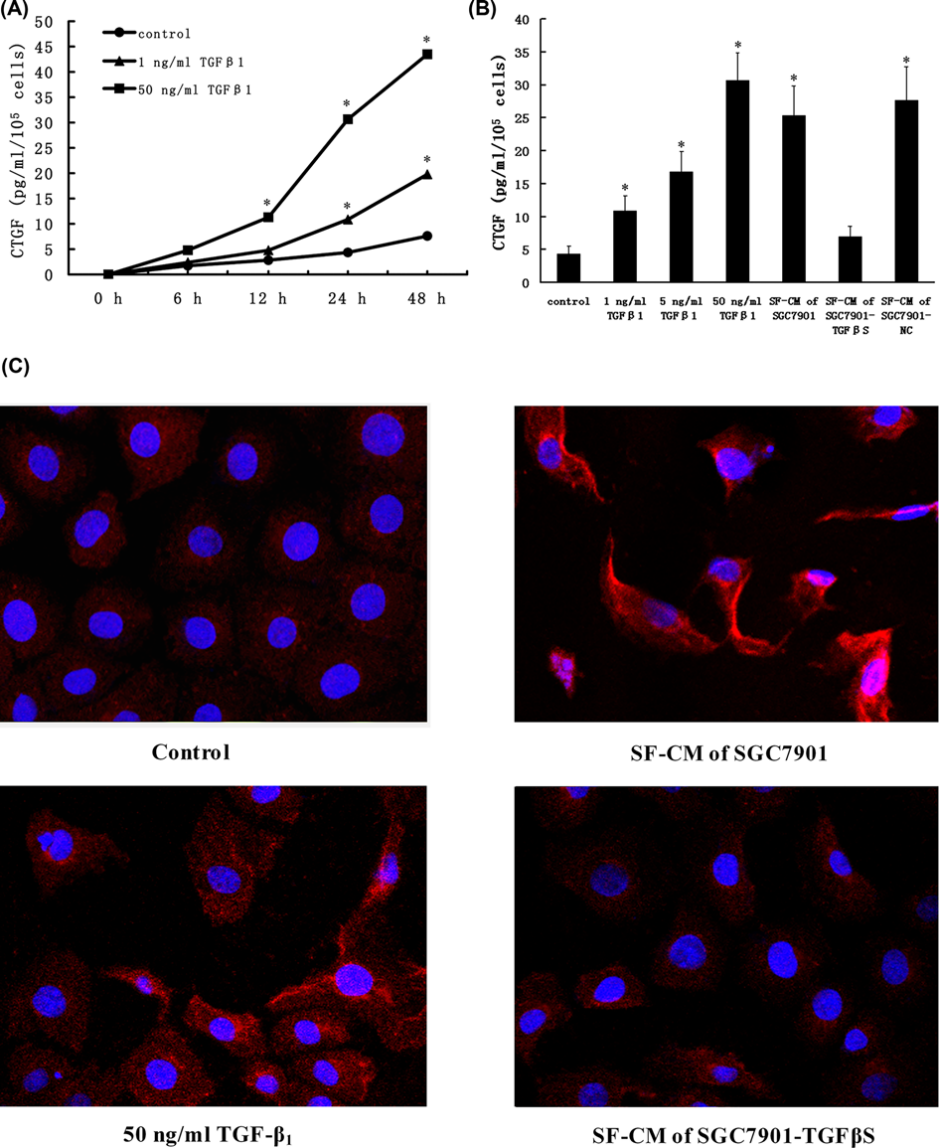
Effect of TGF-β_1_ or SF-CM of gastric cancer cells on CTGF protein production of HPMCs (**A**) HPMCs were cultured for the indicated time in medium containing the indicated concentrations of TGF-β_1_. Each point shows mean ± SD of data from three experiments. (**B**) HPMCs were cultured for 24 h in medium containing various concentrations of TGF-β_1_ or SF-CM of gastric cancer cells. Each column shows mean ± SD of data from three experiments. (**C**) Immunofluorescence analysis of CTGF protein production of HPMCs; red, CTGF; blue, nuclear DNA. *P<0.05 as compared with control (control is 0 ng/ml TGF-β_1_).

Then, TGF-β_1_ was added to HPMCs’ culture and the change of CTGF concentration in the supernatant was examined. The time–response relationship between TGF-β_1_ and CTGF is shown in Figure 3A. When HPMCs were stimulated with 50 ng/ml TGF-β_1_, CTGF was increased at 12 h and later as compaired with control. And TGF-β_1_ produced a time-dependent increase of CTGF. Next, we examined the effects of various concentrations of TGF-β_1_ at the 24 h time point (Figure 3B). TGF-β_1_ produced a dose-dependent increase in CTGF production on HPMCs. Moreover, the SF-CM of SGC7901 was also significantly increased the CTGF concentration of HPMCs, but the SF-CM of SGC7901-TGFβS was not. This was also confirmed by immunofluorescence on confocal microscope (Figure 3C).

### TGF-β_1_ increased CTGF mRNA level

**Figure 4:**
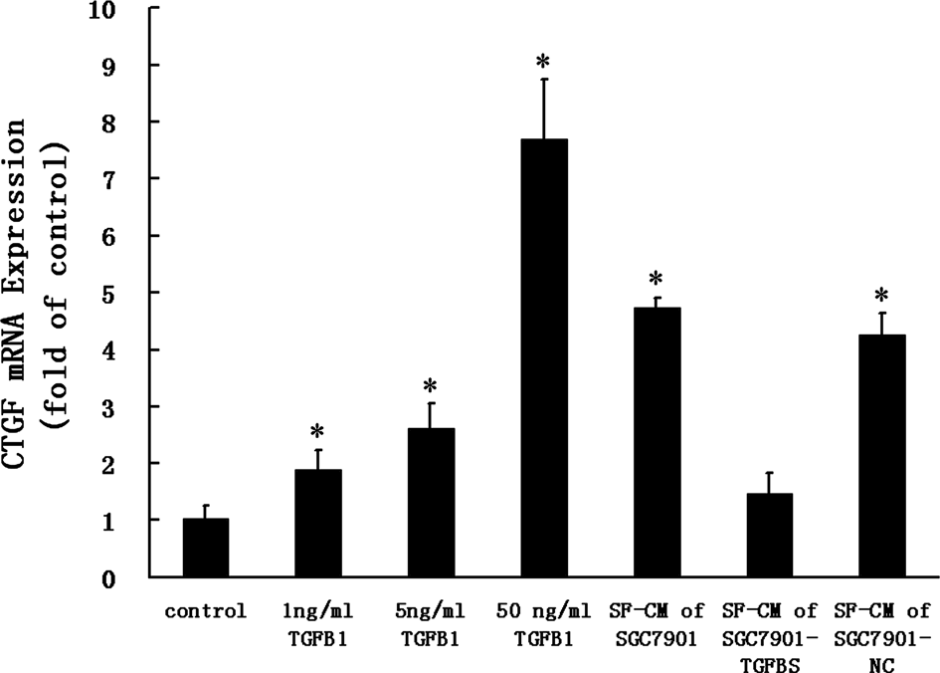
CTGF mRNA expression HPMCs were treated with TGF-β_1_ over a concentration range of 0 to 50 ng/ml or SF-CM of gastric cancer cells for 24 h, and then RT-QPCR was done for detection of CTGF mRNA. Data are expressed as a fold change relative to the untreated control (control is 0 ng/ml TGF-β_1_). Values are given as mean ± SD of three experiments. *P<0.05 as compared with control.

Real-time reverse transcription PCR was done to examine CTGF gene expression. At various concentrations of TGF-β_1_ treatment, there was significant increase of CTGF mRNA expression in a dose-dependent manner. Moreover, incubation of HPMCs with SF-CM of SGC7901 or SGC7901-NC also resulted in a significant increase in CTGF mRNA expression, but the SF-CM of SGC7901-TGFβS did not (Figure 4).

### Morphological alterations of HPMCs after treatment with TGF-β_1_

**Figure 5:**
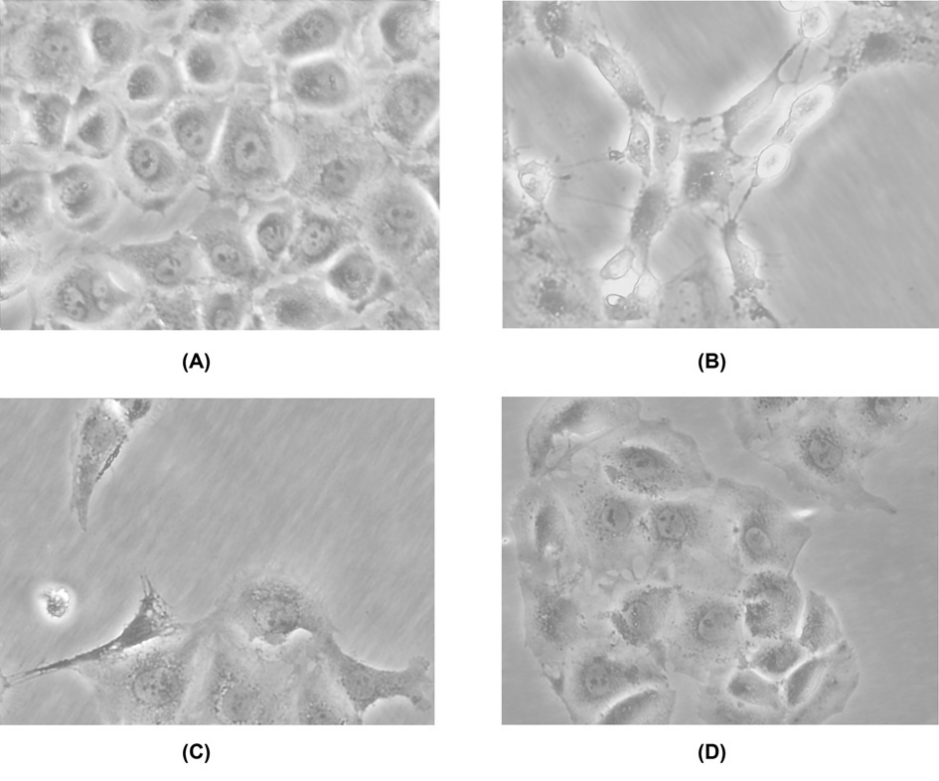
Morphological changes in human peritoneal mesothelial cells under phase contrast microscopy Magnification, ×400. (**A**) Morphology of mesothelial cells cultured in serum-free DMEM. (**B**) Morphological changes in mesothelial cells after incubation with SF-CM of SGC7901. (**C**) Exfoliation and naked areas of mesothelial cells after incubation with 50 ng/ml TGF-β_1_. (**D**) Morphological changes were not obvious in mesothelial cells after incubation with SF-CM of SGC7901-TGFβS.

We observed dramatically different morphology of HPMCs after treatment with TGF-β_1_ or SF-CM of gastric cancer cells. In particular, the non-treated HPMCs exhibited the typical polygonal and cobblestone-like morphology (Figure 5A). In contrast, cells treated with TGF-β_1_ or SF-CM of SGC7901 for 24 h underwent significant morphological alterations. Cells were spindle-like with reduced cytoplasm volume, and with a scattered distribution indicating reduced cell–cell adhesion. Moreover, some cells rounded up and detached from the culture dish, leading to exposed surface area (Figure 5B,C). Interestingly, the above morphological alterations were not obvious after treatment with SF-CM of SGC7901-TGFβS (Figure 5D).

### Induction of apoptosis by TGF-β_1_

**Figure 6:**
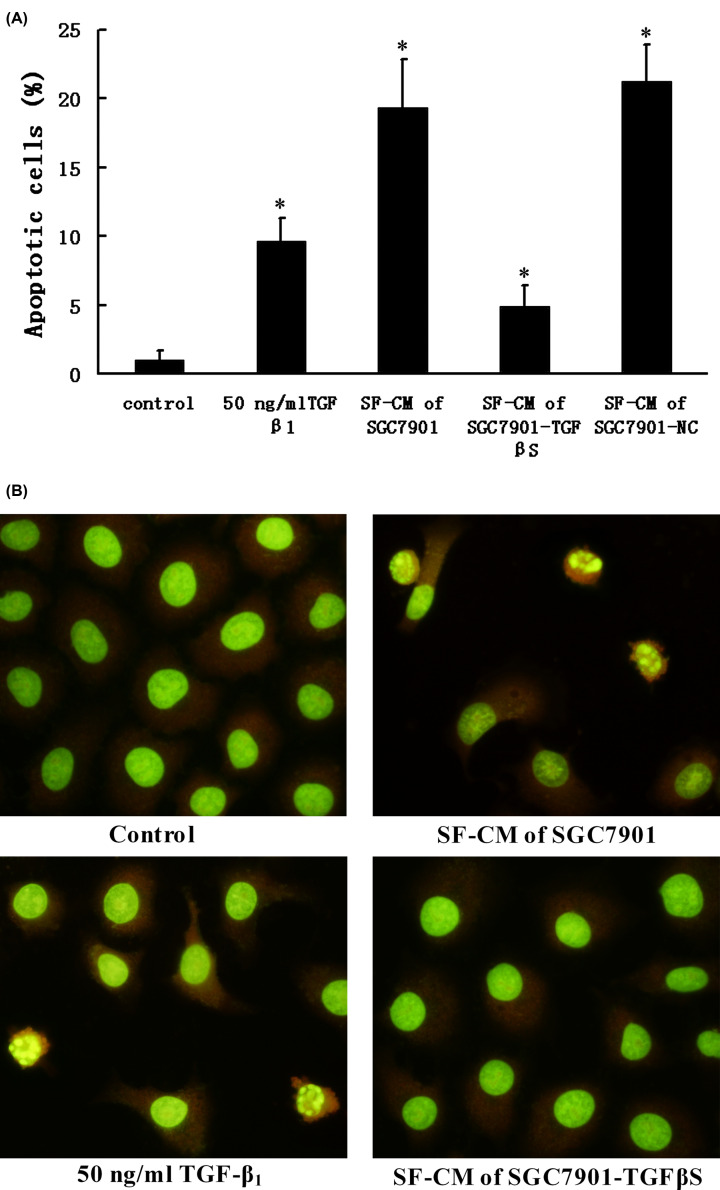
Induction of apoptosis in human peritoneal mesothelial cells The HPMCs were treated with 50 ng/ml TGF-β_1_ or SF-CM of gastric cancer cells for 24 h. (**A**) The cells were collected and stained with propidium iodide for flow cytometry analysis. The results are expressed as mean ± SD of three experiments, *P<0.05 as compared with untreated control (control is 0 ng/ml TGF-β_1_). (**B**) Detection of nuclear fragmentation in apoptotic HPMCs using acridine orange staining. The stained cells were observed under a fluorescent microscope using a blue filter; magnification, ×400.

In order to characterize the effect on apoptosis of HPMCs after treatment with TGF-β_1_ or SF-CM of gastric cancer cells, we analyzed the amount of sub-G_1_ DNA by flow cytometry of fixed nuclei to quantify the degree of apoptosis. As shown in Figure 6A, treatment of HPMCs with 50 ng/ml TGF-β_1_ resulted in an increase in the number of apoptotic cells. The apoptotic rate of HPMCs incubated with SF-CM of SGC7901 and SGC7901-NC was 19.32 ± 3.57% and 21.27 ± 2.63%, respectively. In contrast, the apoptosis rate of HPMCs incubated with SF-CM of SGC7901-TGFβS was significantly lower (5.13 ± 1.53%, *P*<0.05).

We further examined the morphological changes of HPMCs treated with TGF-β_1_ or SF-CM of gastric cancer cells by fluorescence microscopy. The control cells displayed an intact nuclear structure, while cells treated with TGF-β_1_ or SF-CM of SGC7901 had chromosomal condensation and formation of apoptotic bodies (Figure 6B).

### TGF-β_1_ increased gastric cancer cells adhesion to HPMCs

**Figure 7:**
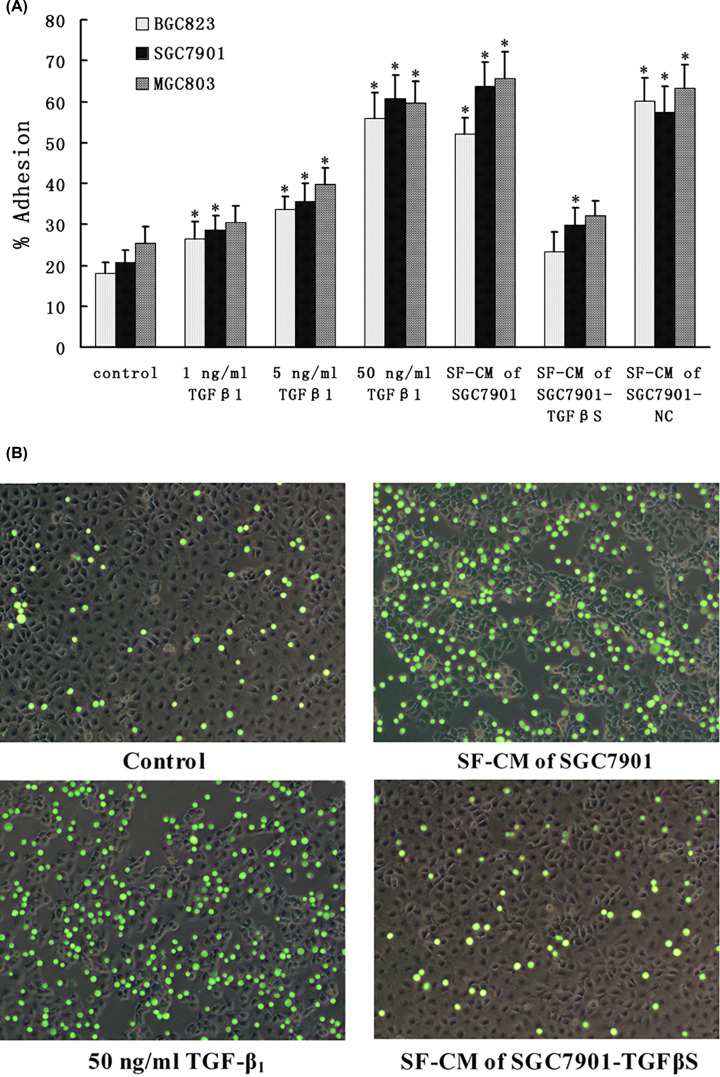
Adhesion of gastric cancer cells to human peritoneal mesothelial cells (**A**) HPMCs were incubated with TGF-β_1_ or SF-CM of gastric cancer cells for 24 h prior to the addition of gastric cancer cells. Then, fluorescently labeled gastric cancer cells were overlaid on HPMCs and incubated at 37°C for 2 h. After gentle washing to remove nonadherent cells, the fluorescence was measured with a fluorescent plate reader (EX = 485 nm, EM = 530 nm). Bars represent the mean ± SD of three experiments. *P<0.05 as compared with control (control is 0 ng/ml TGF-β_1_). (**B**) The adhesion of fluorescently labeled gastric cancer cells to HPMCs was observed by fluorescent microscope. Gastric cancer cells containing CFSE exhibit green staining. The background cells are HPMCs; magnification, ×100.

To determine the effect of TGF-β_1_ on cancer–mesothelial cell adhesion, a monolayer of HPMCs was incubated with TGF-β_1_ or SF-CM of gastric cancer cells for 24 h. Then, fluorescence labeled gastric cancer cells were added to the monolayer of HPMCs for another 2 h. After rinsing away the non-adherent gastric cancer cells from the co-culture, the fluorescence intensity was measured to represent the remaining adherent gastric cancer cells on HPMCs. We found that TGF-β_1_ could promote the adhesion of gastric cancer cells to HPMCs in a dose-dependent manner (Figure 7A). We also found that more gastric cancer cells were able to adhere to mesothelial cell monolayer after treatment with SF-CM of SGC7901 or SGC7901-NC. In contrast, we didn’t find significant increase of the adhesion to HPMCs after treatment with SF-CM of SGC7901-TGFβS. This was also confirmed by fluorescence imaging (Figure 7B).

### Down-regulation of TGF-β_1_ inhibited peritoneal metastasis of gastric cancer and decreased CTGF expression *in vivo*

**Figure 8:**
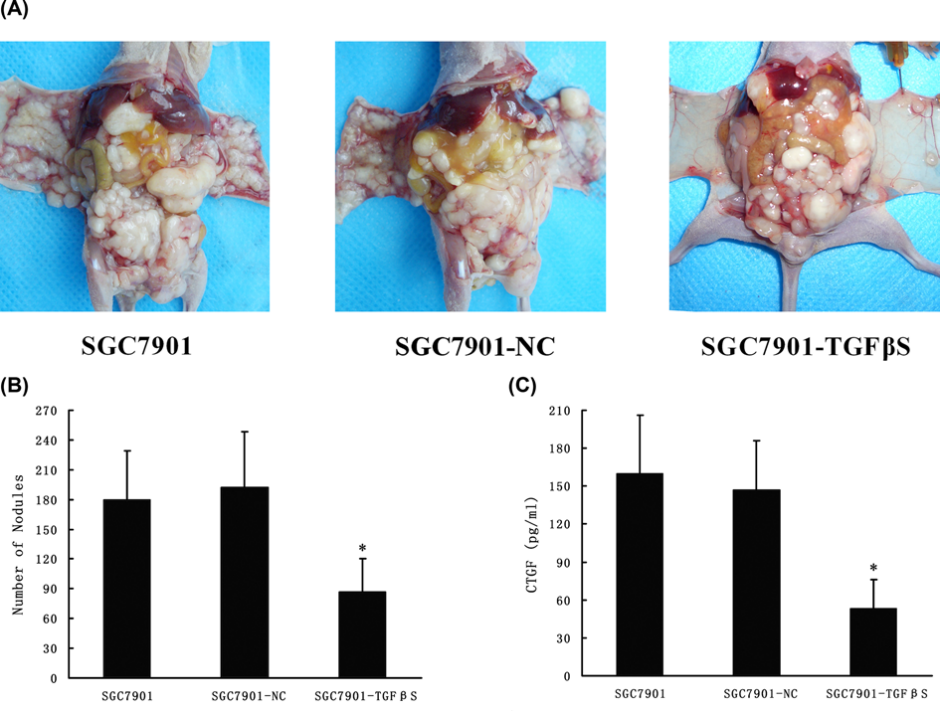
Knockdown of TGF-β_1_ inhibited gastric cancer SGC7901 xenograft peritoneal metastasis and decreased CTGF expression *in vivo* SGC7901, SGC7901-NC and TGF-β_1_ knockdown stable cell line SGC7901-TGFβS were injected intraperitoneally as described under ‘Material and Methods’ section. Seven weeks later, the mice were killed, photographed, dissected and any disseminated nodules present on the mesentery and diaphragm were counted. (**A**) The photograph of nude mice with peritoneal dissemination from each group. (**B**) The disseminated nodules were evaluated. (**C**) Ascites was collected from peritoneal cavity. CTGF protein in the ascites was quantified by ELISA method. Each bar represents the mean ± SD (*n*=10 for each group). *P<0.05 as compared with control (control is SGC7901).

To explore the effects of TGF-β_1_ on the peritoneal metastasis of gastric cancer *in vivo*, we inoculated different transfectants into nude mice. SGC7901 cells, control stable cells (SGC7901-NC) and TGF-β_1_ knockdown stable cells (SGC7901-TGFβS) were injected into three separate groups of nude mice. As a consequence of such treatment, the apparent suppression of peritoneal dissemination in mice injected with SGC7901-TGFβS cells as compared with those injected with SGC7901 cells or SGC7901-NC cells was noted (Figure 8A). Quantitatively, 180 ± 49 disseminated nodules were noted for mice inoculated with SGC7901 cells and 192 ± 56 disseminated nodules were noted for mice inoculated with SGC7901-NC cells. In contrast, significant fewer disseminated nodules were able to be observed for mice injected with SGC7901-TGFβS cells (85 ± 33; Figure 8B). Then, we investigated the level of CTGF protein in ascites by ELISA method. The level of CTGF in the ascites of nude mice inoculated with SGC7901-TGFβS cells was significantly lower than the control cell lines (Figure 8C).

## Discussion

Peritoneal metastasis is not only the most frequent pattern of gastric cancer recurrence, but also a major cause of death among patients of advanced gastric cancer [20]. Although the presence of peritoneal metastasis reveals a strong impact for patient prognosis, the molecular mechanisms by which gastric cancer cells actually acquire the ability to undergo peritoneal dissemination remain to be clarified.

Recent years, various researchers have examined the biochemical network of growth factors interactions in gastric cancer. However, a few of them examined seem to be suitable diagnostic biomarkers for detecting gastric cancer in humans [21–23]. Data from one multicenter transcriptome study and The Cancer Genome Atlas established the significance of TGF-β_1_ signaling on gastric cancer progression, supporting its role as an emerging candidate biomarker for gastric cancer [24,25]. In line with these pivotal studies, others also showed that gastric cancer patients with high expression of TGF-β_1_ had unfavorable prognosis [26–28].

TGF-β_1_ is a polypeptide with a molecular weight of 25,000 DA and TGFβs have been cloned as three types in mammals: TGF-β_1_, TGF-β_2_ and TGF-β_3_ [29]. TGF-β_1_ plays an important role in the maintenance of homeostasis in various organs, including the gastric epithelium [30]. Recently, TGF-β_1_ has been studied in functions such as the regulation of cell proliferation, cell differentiation, apoptosis, protein synthesis, analysis of extracellular matrix, and expression of adhesion molecules [5,31]. In oncogenesis, TGF-β_1_ functions in both tumor suppression and tumor promoting activities, depending on the stage of cancer and the responsivity of the cancer cell. In early tumor stages, TGF-β_1_ is tumor suppressive, and in advanced tumor stages, it is tumor promoting [8,9]. It has been reported that various cancers are associated with TGF-β_1_ [32–35]_._ However, whether TGF-β_1_ influences the peritoneal metastasis of gastric cancer remains unknown.

In the present study, we observed under phase contrast microscope that mesothelial cells became hemispherical and exfoliation occurred when TGF-β_1_ was added into HPMCs’ culture. Furthermore, both fluorescent microscopy and flow cytometry analysis confirmed that apoptosis of HPMCs was significantly increased in response to TGF-β_1_ treatment. It has been previously reported that healthy mesothelial cells can prevent cancer cells adhesion and invasion by providing an intact barrier. Cancer cells are also known to secrete various factors to induce damages or apoptosis in mesothelial cells and the disrupted mesothelial cells may become prone to the invasion of cancer cells [36,37]. Therefore, the morphological alterations and apoptosis of mesothelial cells may function as a favorable environment for peritoneal metastasis. To investigate whether the induction of TGF-β_1_ could enhance tumor–mesothelial adhesion, we incubated the HPMCs with different concentrations TGF-β_1_. Then we added gastric cancer cells for another 2 h to evaluate the adhesion ability to mesothelial cells under various conditions. As a consequence of such treatment, TGF-β_1_ increased tumor–mesothelial adhesion in a dose-dependent manner. Interestingly, these findings were also occurred by treatment with SF-CM of gastric cancer cell line SGC7901 that produced a large amount of TGF-β_1_. However, the effects of morphological alteration, apoptosis and increase in adhesion were significantly decreased when HPMCs were treated with SF-CM of SGC7901-TGFβS, a TGF-β_1_ knockdown stable cell line. Since tumor–mesothelial adhesion is one of the critical steps of peritoneal dissemination, we further investigated the effects of TGF-β_1_ on peritoneal metastasis of gastric cancer cells *in vivo*. As shown, significant fewer disseminated nodules were observed for mice injected with SGC7901-TGFβS cells as compared with those injected with SGC7901 cells or SGC7901-NC cells. Taken together, these data demonstrate that TGF-β_1_ is critically involved in the peritoneal metastasis of gastric cancer and can promote peritoneal metastasis.

CTGF is a novel, potent angiogenic factor, which was first defined as a mitogen, detected in conditioned medium from human umbilical vein endothelial cells [38]. Integrin is an important receptor for CCN proteins, and receptor activation may produce a variety of effects. CTGF protein can bind directly to integrins α_V_β_3_ and α_IIb_β_3_ [39]. CTGF plays a role in the development and progression of cancer. Yang et al. reported that CTGF is a downstream mediator of TGF-β_1_ action in cancer associated reactive stroma, one of the key promoters of angiogenesis in tumor-reactive stromal microenvironment, and plays an important role in prostate carcinogenesis [40]. However, the interaction between TGF-β_1_ and CTGF in the progression of peritoneal metastasis has not been examined.

In the present study, we found that the CTGF concentrations of HPMCs were significantly elevated after incubated with TGF-β_1_ or SF-CM of SGC7901 that produced a large amount of TGF-β_1_. And TGF-β_1_ was also shown to induce CTGF mRNA expression. In contrast, the CTGF protein or mRNA didn’t significantly change after incubation with SF-CM of SGC7901-TGFβS, a TGF-β_1_ knockdown stable cell line. Furthermore, the level of CTGF protein in the ascites of nude mice inoculated with SGC7901-TGFβS cells was significantly lower than the control cell lines. These results indicate that TGF-β_1_ can induce CTGF secretion of peritoneal mesothelial cells in the progression of peritoneal metastasis.

In conclusion, our present findings indicated that TGF-β_1_ could induce CTGF expression and apoptosis of HPMCs, increase gastric cancer cells adhesion to HPMCs and promote peritoneal metastasis of gastric cancer. Our study provides evidence that peritoneal mesothelial cells play important roles in the development of peritoneal dissemination. Novel methods targeting TGF-β_1_ signaling pathways might be effective to prevent and treat peritoneal metastasis, although additional studies are needed.

## Abbreviations

CTGF: connective tissue growth factor
HPMC: human peritoneal mesothelial cell
SF-CM: serum-free conditional medium
siRNA: small interfering RNA
TGF-β_1_: transforming growth factor-β_1_
